# Systematic pan-cancer analysis showed that RAD51AP1 was associated with immune microenvironment, tumor stemness, and prognosis

**DOI:** 10.3389/fgene.2022.971033

**Published:** 2022-11-16

**Authors:** Renwang Liu, Guangsheng Zhu, Mingbiao Li, Peijun Cao, Xuanguang Li, Xiuwen Zhang, Hua Huang, Zuoqing Song, Jun Chen

**Affiliations:** ^1^ Department of Lung Cancer Surgery, Lung Cancer Institute, Tianjin Medical University General Hospital, Tianjin, China; ^2^ Tianjin Key Laboratory of Lung Cancer Metastasis and Tumour Microenvironment, Lung Cancer Institute, Tianjin Medical University General Hospital, Tianjin, China

**Keywords:** RAD51AP1, carcinogenesis, tumor immune microenvironment, pan-cancer, bioinformatics analysis

## Abstract

Although RAD51 associated protein 1 (RAD51AP1) is crucial in genome stability maintenance, it also promotes cancer development with an unclear mechanism. In this study, we collected intact expression data of RAD51AP1 from the public database, and verified it was significantly over-expressed in 33 cancer types and correlated with poor prognosis in 13 cancer types, including glioma, adrenocortical carcinoma, lung adenocarcinoma. We further authenticated that RAD51AP1 is up-regulated in several typical cancer cell lines and promotes cancer cell proliferation *in vitro*. Moreover, we also demonstrated that RAD51AP1 was significantly positively related to cancer stemness score mRNAsi in 27 cancer types and broadly correlated to tumor-infiltrating immune cells in various cancers in a diverse manner. It was also negatively associated with immunophenoscore (IPS) and Estimation of STromal and Immune cells in MAlignant Tumours using Expression data (ESTIMATE) scores and positively correlated with mutant-allele tumor heterogeneity (MATH), tumor mutational burden (TMB), microsatellite instability (MSI), and PD-L1 expression in multiple cancers. The tumor stemness enhancing and tumor immune microenvironment affecting functions of RAD51AP1 might compose its carcinogenesis mechanism. Further investigations beyond the bioinformatics level should confirm these findings in each specific cancer.

## Introduction

Homologous recombination (HR) is critical in genome maintenance and tumorigenesis suppression (Moynahan and Jasin, 2010). RAD51 associated protein 1 (RAD51AP1) promotes HR by interacting with recombinase RAD51 and stimulating its mediated D-loop formation (Wiese et al., 2007). RAD51AP1 also enhances meiotic HR through binding to DNA meiotic recombinase 1 (DMC1) (Dray et al., 2011). This evidence demonstrated that RAD51AP1 is crucial in maintaining cellular genome homeostasis. However, recent studies have shown that RAD51AP1 was highly expressed in several tumor tissues, and its high expression also indicated a poor prognosis (Pathania et al., 2016; Li et al., 2018a; Le et al., 2019; Bridges et al., 2021). Vitro and vivo experiments also confirmed that RAD51AP1 promotes cancer cell proliferation, invasion, and migration and inhibits apoptosis (Obama et al., 2008; Chudasama et al., 2018; Wu et al., 2019). Thus, although RAD51AP1 plays a vital role in genome homeostasis maintenance, it may otherwise act as an oncogene in many organs.

The mechanism of RAD51AP1 in promoting tumorigenesis and cancer development is still unclear. Cancer stemness maintenance and promotion may count as the leading cause (Bridges et al., 2020). However, this mechanism was only obtained in breast cancer and colorectal cancer (CRC), and it is still unclear whether it has the same effect in other cancers (Bridges et al., 2020; Bridges et al., 2021). Meanwhile, the effect of RAD51AP1 on immune cell infiltration in the tumor microenvironment and its correlation with tumor heterogeneity, microsatellite instability (MSI), tumor mutational burden (TMB), and RNA modification have not been reported yet.

Therefore, we conducted a data mining investigation on the public database by multiple bioinformatics methods in this paper. We analyzed the differential expressions of RAD51AP1 in pan-cancer and explored its correlation with prognosis, tumor stemness, RNA modification, tumor immunity, and tumor heterogeneity, aiming to preliminary explore the potential mechanism of RAD51AP1 in cancer development.

## Materials and methods

**FIGURE 1:**
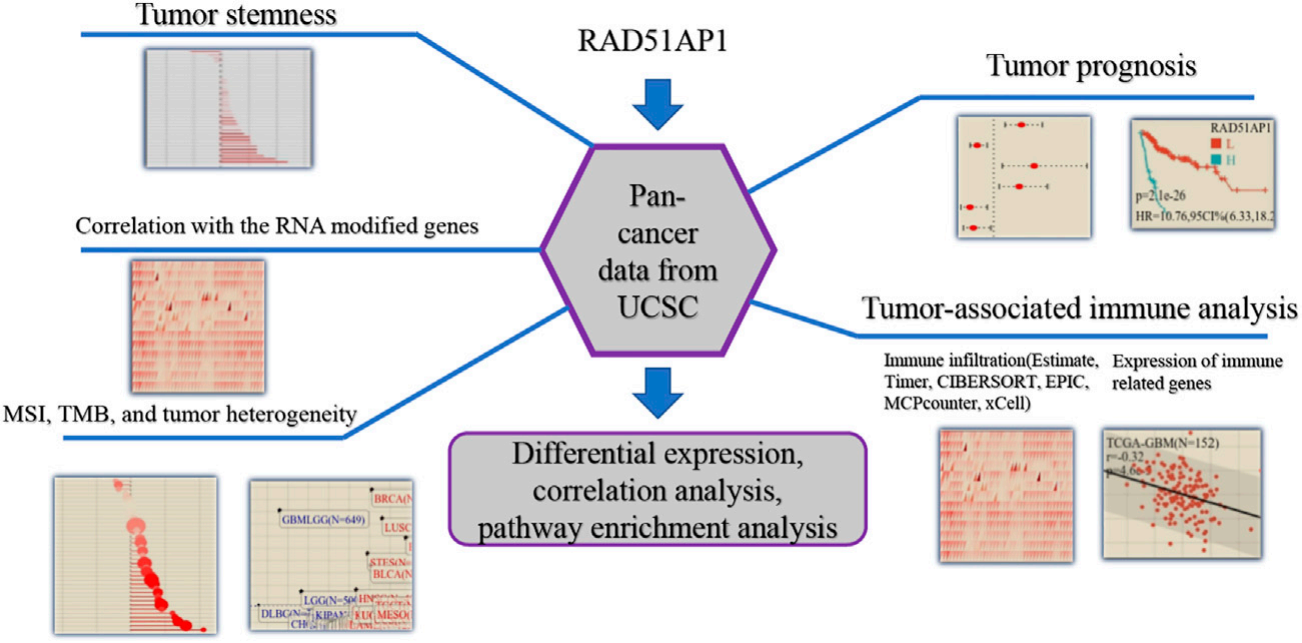
Flowchart of the principal bioinformatics analysis in this study.

The flowchart of the bioinformatic analysis in this study is shown in Figure 1. The specific details of all the methods are as follows.

### Gene expression data acquisition

Unified pan-cancer datasets were downloaded from The Cancer Genome Atlas (TCGA) and Therapeutically Applicable Research to Generate Effective Treatments (TARGET) databases, and normal tissue datasets were downloaded from Genotype-Tissue Expression Project (GTEx) as control (PANCAN, *N* = 19131, *G* = 60499). RAD51AP1 (ENSG00000111247) gene expression data were extracted from each sample. Samples from Primary Blood Derived Cancer - Peripheral Blood (TCGA-acute myeloid leukemia (LAML)), Primary Tumor, Metastatic of TCGA-skin cutaneous melanoma (SKCM), Primary Blood Derived Cancer—Bone Marrow, Primary Solid Tumor and Recurrent Blood Derived Cancer—Bone Marrow were selected subsequently. All expression values were log2 (x+0.001) transformed. Differential expression analysis and clinical feature analysis were performed in R software (version 3.6.4). The abbreviation of each cancer type was listed in Table 1.

**TABLE 1:** Abbreviation of each cancer type.

Abbreviation	Term
ACC	Adrenocortical carcinoma
ALL	Acute lymphoblastic leukemia
ALL-R	Recurrent acute lymphoblastic leukemia
BLCA	Bladder urothelial carcinoma
BRCA	Breast invasive carcinoma
CESC	Cervical squamous cell carcinoma and endocervical adenocarcinoma
CHOL	Cholangiocarcinoma
COAD	Colon adenocarcinoma
COADREAD	Colon adenocarcinoma/rectum adenocarcinoma
DLBC	Lymphoid neoplasm diffuse large B-cell lymphoma
ESCA	Esophageal carcinoma
GBM	Glioblastoma multiforme
GBMLGG	Glioma
HNSC	Head and neck squamous cell carcinoma
KICH	Kidney chromophobe
KIPAN	Pan-kidney cohort (KICH+KIRC+KIRP)
KIRC	Kidney renal clear cell carcinoma
KIRP	Kidney renal papillary cell carcinoma
LAML	Acute myeloid leukemia
LGG	Brain lower grade glioma
LIHC	Liver hepatocellular carcinoma
LUAD	Lung adenocarcinoma
LUSC	Lung squamous cell carcinoma
MESO	Mesothelioma
NB	Neuroblastoma
OS	Osteosarcoma
OV	Ovarian serous cystadenocarcinoma
PAAD	Pancreatic adenocarcinoma
PCPG	Pheochromocytoma and paraganglioma
PRAD	Prostate adenocarcinoma
READ	Rectum adenocarcinoma
SARC	Sarcoma
SKCM	Skin cutaneous melanoma
SKCM-M	Metastatic skin cutaneous melanoma
SKCM-P	Primary skin cutaneous melanoma
STAD	Stomach adenocarcinoma
STES	Stomach and esophageal carcinoma
TGCT	Testicular germ cell tumors
THCA	Thyroid carcinoma
THYM	Thymoma
UCEC	Uterine corpus endometrial carcinoma
UCS	Uterine carcinosarcoma
UVM	Uveal melanoma
WT	High-risk wilms tumor

### Survival analysis

A high-quality TCGA prognostic dataset was downloaded from a TCGA-based study (Liu et al., 2018a). Follow-up data shorter than 30 days was downloaded from TARGET. Cancer types with less than ten samples were excluded, and 44 cancer types with overall survival (OS) data and 38 cancer types with progression-free survival (PFS) data were finally collected. Cox proportional hazards regression model was established by CoxPH in R software. MaxStat in R software was used to calculate the best cut-off value of RAD51AP1 expression by setting 25%–75% as the grouping number range. After dividing each cancer type into high and low RAD51AP1 expression groups, we used survfit in R to analyze the differences in OS and PFS between the two groups.

### Expression correlation analysis

All the level 4 Simple Nucleotide Variation datasets in TCGA, which were processed by MuTect2 software, were downloaded from Genomic Data Commons (GDC) (https://portal.gdc.cancer.gov/) (Beroukhim et al., 2010). The mutation and expression data were integrated, and the synonymous mutations data were filtered subsequently. Cancer types that had less than three samples were excluded. We further analyzed the correlation between the expression of RAD51AP1 and other genes and performed enrichment analysis *via* Gene Ontology (GO) and Kyoto Encyclopedia of Genes and Genomes (KEGG) pathway analysis. Meanwhile, the expression data of immune pathway marker genes, immune checkpoint genes, and all 44 RNA medication marker genes, including m1A, m5C, and m6A, were also collected and analyzed in each tumor sample, exclusively.

### Immune cell infiltration and immune score analysis

Six independent tumor-infiltrating immune cells (TICs) analysis methods from the R package IOBR (version 0.99.9) (Zeng et al., 2021) including TIMER (Li et al., 2017), deconvo_EPIC (Racle et al., 2017), deconvo_MCPcounter (Becht et al., 2016), deconvo_xCell (Aran et al., 2017), deconvo_CIBERSORT (Newman et al., 2015) and deconvo_quanTIseq (Finotello et al., 2019) were performed in this study. We also investigated the correlation between RAD51AP1 expression and tumor-associated immune comprehensive score *via* deconvo_ips in IOBR and Estimation of STromal and Immune cells in MAlignant Tumours using Expression data (ESTIMATE) in R (Yoshihara et al., 2013; Charoentong et al., 2017). All 44 cancer types and 10180 samples were available in EPIC, MCPcounter, xCell, CIBERSORT, quanTIseq, immunophenoscore (IPS), and ESTIMATE score method, and with it 38 cancer types and 9406 samples available in TIMER.

### Tumor heterogeneity, TMB, MSI, and stemness scoring

Mutation data were collected the same as previously mentioned. Mutant-allele tumor heterogeneity (MATH) and TMB were calculated by inferHeterogeneity and tmb function from the R package maftools (version 2.8.05). MSI was calculated using the method reported by *R.* Bonneville et al. (2017). The stemness score was evaluated using the stemness scoring algorithm developed by T.M. Malta et al. (2018), which calculates the mRNAsi score through the mRNA signature and the mDNAsi score through the methylation signature. Cancer types with less than three samples were also excluded in this part.

### Cell culture and cell transfection

All the cell lines in this study were purchased from American Tissue Culture Collection (ATCC). OVCAR3, Hep3B, PANC1, H1975, A549, and BEAS-2B were maintained in RPMI 1640 medium (Gibco, United States). MCF-7 and THLE-3 were maintained in DMEM (Gibco, United States) and BEGM (Lonza, United States), respectively. The BEGM was supplemented with 10% fetal bovine serum (FBS), phosphoethanolamine (70 ng/ml), and epidermal growth factor (EGF) (5 ng/ml). The rest culture media were supplemented with 10% FBS. The plasmids were obtained from Biomed Gene Technology Co., LTD (Beijing, China). The pCDNA3.1-RAD51AP1 or pCDNA3.1 encoding nonspecific sequence was transfected into each type of cancer cells by Lipofectamine 2000 (Invitrogen, United States), respectively.

### Cell counting Kit-8 assay and colony formation assay

Cells were suspended and seeded in 96-well plates at 4000 cells per well. After 24h, 48h, and 72 h incubation, 10 μl CCK8 (APExBIO, United States) was added to each well and incubated for 1 h. Afterward, the OD value at 450 nm was measured using a microplate reader. In the colony formation assay, cells were seeded in 6-well plates at 400 cells per well and cultured for 10–14 days until naked-eye visible clones appeared. 4% paraformaldehyde was used to fix cells for 15 min , and then cells were stained with 0.1% crystal violet for 30 min . Afterward, cells were imaged, and the number of colonies was counted.

### Western blot and qPCR

Western blot was performed as previously described (Li et al., 2018b). Primary antibodies used were: anti- RAD51AP1 (1:1000, Proteintech, China) and anti-α Tubulin (1:5000, Abcam, United Kingdom). Real-time PCR was performed to evaluate the plasmid’s transfection effectiveness. Briefly, total RNA was extracted by TRIzol reagent (Invitrogen, United States), and cDNA were synthesized using PrimeScript RT Reagent Kit (TaKaRa, China). Each sample was tested in triplicate, and results were normalized by qPCR of cDNA with β-actin. The RAD51AP1 forward primer was designed as ATG​ACA​AGC​TCT​ACC​AGA​GAG​AC, and the β-actin forward primer was TCG​TGC​GTG​ACA​TTA​AGG​AGA​AGC.

### Statistical analysis

Statistical analysis was performed by R software (version 3.6.4). Unpaired data were analyzed by Wilcoxon Rank Sum and Signed Rank Tests. Samples with multiple groups were analyzed by the Kruskal test. Pearson’s correlation coefficient was used for the correlation analysis, and the Log-rank test was used for survival analysis. *p*-value ≤0.05 was considered significant.

## Results

### RAD51AP1 was significantly overexpressed in most tumors and occasionally positively correlated with malignant clinical features

**FIGURE 2:**
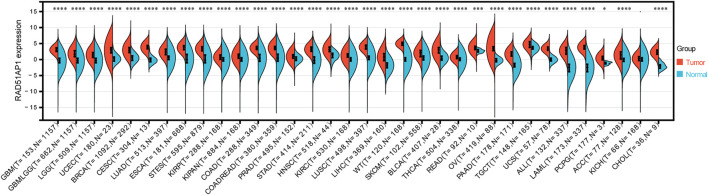
Differential expression analysis of RAD51AP1 in pan-cancer. All available expression data in 34 cancer types were performed differential expression analysis. Thirty-there cancer types including GBM(*p =* 2.2e-88), GBMLGG (*p* = 1.4e-189), LGG (*p =* 2.9e-141), UCEC (*p =* 2.0e-10), BRCA (*p =* 2.1e-121), CESC(*p =* 2.3e-9), LUAD (*p =* 9.1e-89), ESCA(*p =* 7.7e-86), STES (*p =* 1.8e-198), KIRP (*p =* 1.2e-12), KIPAN (*p =* 1.9e-30), COAD (*p =* 1.4e-98), COADREAD (*p =* 1.2e-112), PRAD (*p =* 1.3e-17), STAD (*p =* 7.5e-85), HNSC(*p =* 3.2e-20), KIRC (*p =* 7.3e-42), LUSC(*p =* 1.6e-141), LIHC(*p =* 3.6e-44), WT (*p =* 2.3e-47), SKCM(*p =* 9.0e-23), BLCA (*p =* 5.9e-12), THCA(*p =* 3.8e-34), READ (*p =* 1.9e-4), OV(*p =* 2.6e-48), PAAD (*p =* 6.5e-53), TGCT (*p =* 1.9e-18), UCS(*p =* 5.3e-23), ALL (*p =* 6.0e-60), LAML (*p =* 5.1e-76) and PCPG (*p =* 0.02) presented significantly overexpressed RAD51AP1 in tumor tissues.

Thirty-three of 34 cancer types presented RAD51AP1 significantly up-regulated in tumor samples, including glioblastoma multiforme (GBM), brain lower grade glioma (LGG), uterine corpus endometrial carcinoma (UCEC), breast invasive carcinoma (BRCA), and lung adenocarcinoma (LUAD) (Figure 2). Meanwhile, in cervical squamous cell carcinoma and endocervical adenocarcinoma (CESC), colon adenocarcinoma (COAD), and colon adenocarcinoma/rectum adenocarcinoma (COADREAD), the expression of RAD51AP1 was significantly negatively correlated with age (Supplementary Figure S1A). Male patients in LAML, thymoma (THYM), and mesothelioma (MESO) presented significantly higher RAD51AP1 expression than females (Supplementary Figure S1B). Increased RAD51AP1 expression also indicated high-grade differentiation in stomach and esophageal carcinoma (STES), kidney renal papillary cell carcinoma (KIRP), and UCEC, higher T stage in pancreatic adenocarcinoma (PAAD) and uterine carcinosarcoma (UCS), higher M stage in LGG, and MESO, and higher TNM stage in UCEC and thyroid carcinoma (THCA) (Supplementary Figures S1C–F).

### Up-regulated RAD51AP1 usually indicated a poor prognosis

**FIGURE 3:**
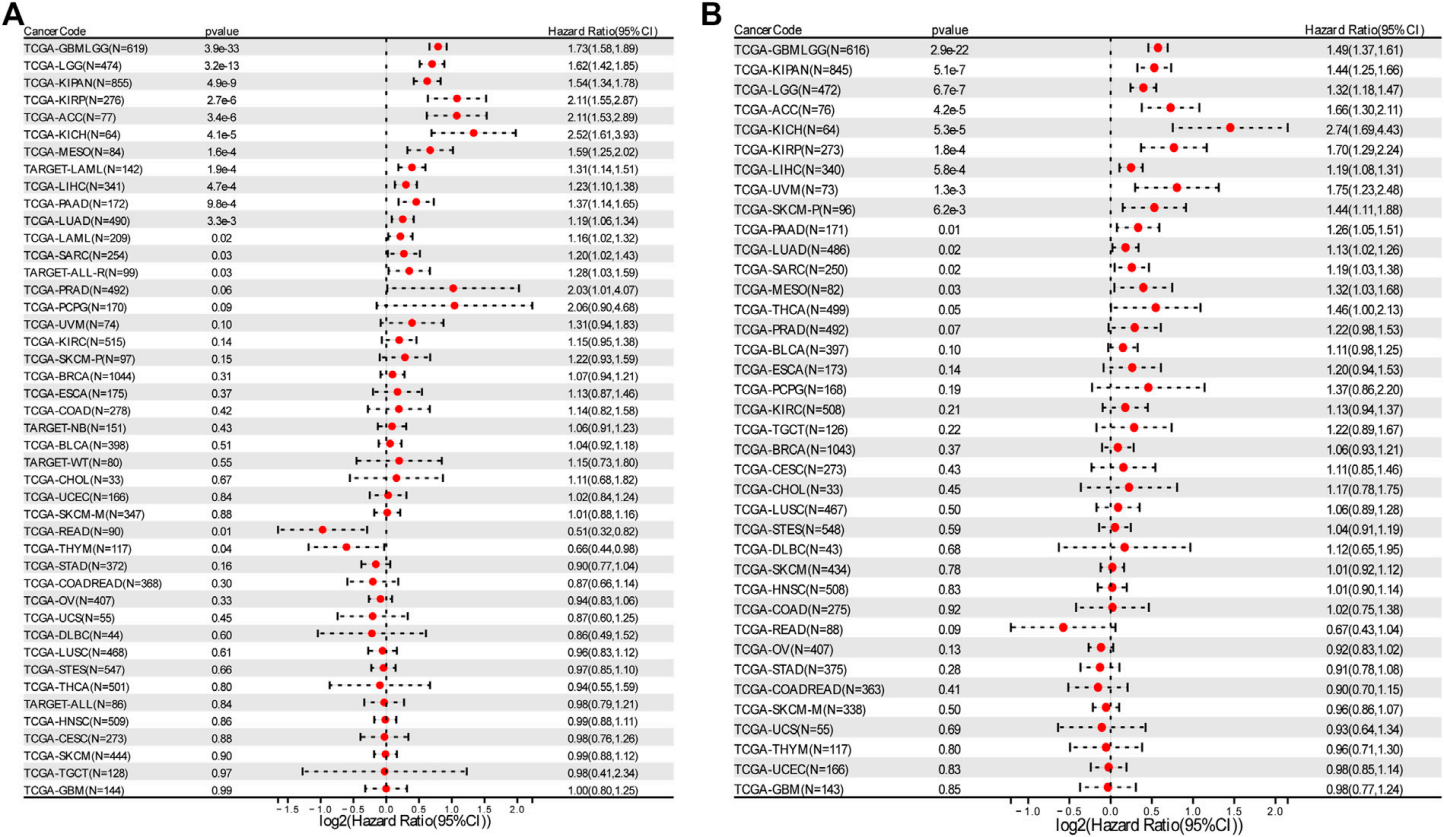
Survival analysis of RAD51AP1 in pan-cancer. **(A)** 43 cancer types were analyzed using R software’s coxph function. High expression RAD51AP1 significantly correlated to the poor OS in GBMLGG, LGG, LAML (from TCGA and TARGET database), LUAD, SARC, KIRP, KIPAN, LIHC, MESO, PAAD, ACC, ALL-R, and KICH. Conversely, in READ and THYM, low expression of RAD51AP1 is significantly linked to poor prognosis. **(B)** PFS analysis of 38 cancer types showed poor prognosis of GBMLGG, LGG, LUAD, SARC, KIRP, KIPAN, LIHC, SKCM-P, MESO, UVM, PAAD, ACC, and KICH related to highly expressed RAD51AP1.

Overall survival analysis showed that higher expression of RAD51AP1 correlated to worse prognosis in LGG, KIRP, LAML, LUAD, *etc.* 13 cancer types and better prognosis in only two cancer types, THYM, and rectum adenocarcinoma (READ) (Figure 3A). Meanwhile, progression-free interval analysis showed that highly expressed RAD51AP1 was also related to poor prognosis in 13 cancer types, including LGG, KIRP, LUAD, and sarcoma (SARC) (Figure 3B). These correlations were reconfirmed using the Log-rank test (shown in Supplementary Figures S2,S3 for OS and PFS, respectively).

### RAD51AP1 expression might be correlated with cell cycle and p53 pathways

**FIGURE 4:**
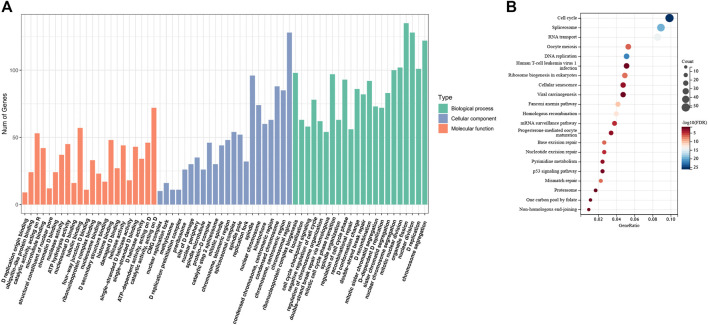
Enrichment analysis of the genes correlated expressed with RAD51AP1. **(A)** Gene Ontology (GO) enrichment analysis. **(B)** Kyoto Encyclopedia of Genes and Genomes (KEGG) pathway analysis.

We collected all the genes that correlated expressed with RAD51AP1 (shown in Supplementary Table S1). Subsequently, GO enrichment analysis showed that expression of RAD51AP1 was mainly correlated with HR and DNA damage repair-associated signaling pathways (Figure 4A). It was also significantly correlated with the cell cycle checkpoint signaling pathway (Figure 4A). The KEGG signaling pathway showed a significant correlation between RAD51AP1 expression and the p53 signaling pathway (Figure 4B).

### RAD51AP1 was generally positive related to tumor stemness

**FIGURE 5:**
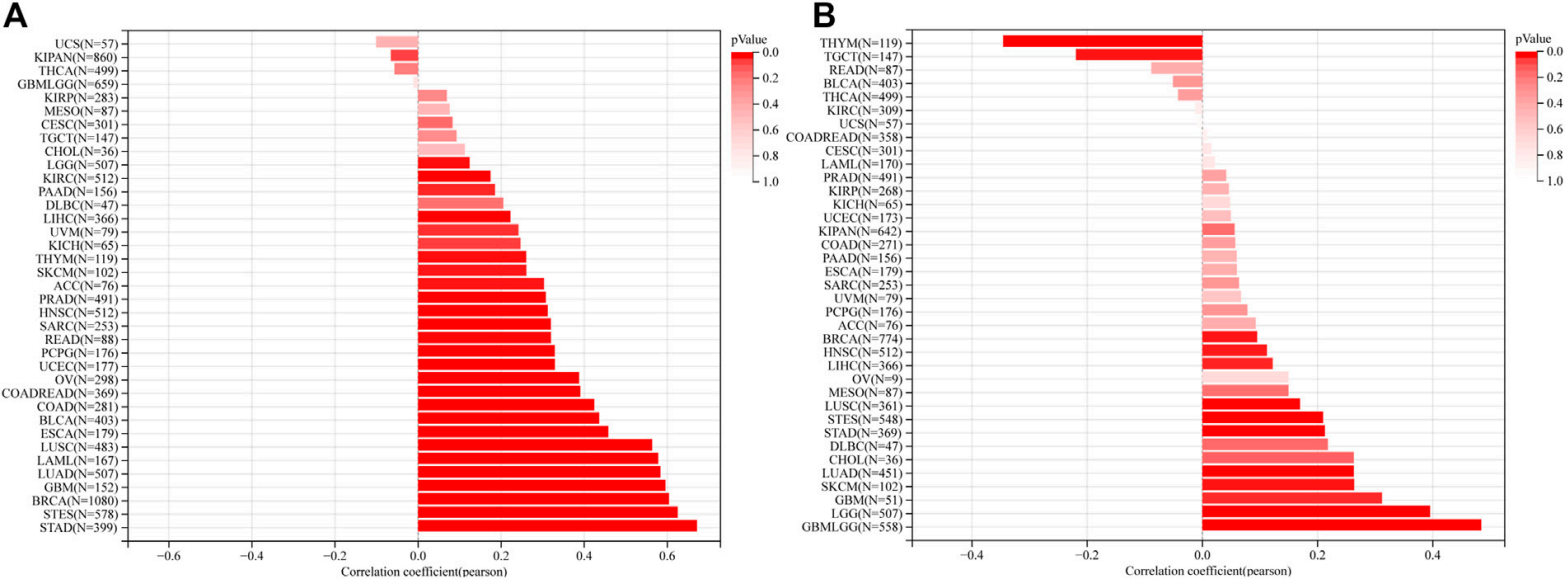
Correlation between RAD51AP1 expression and cancer stemness. **(A)** mRNAsi scoring analysis showed that RAD51AP1 expression positively related to cancer stemness in 27 cancer types. **(B)** mDNAsi scoring analysis showed that RAD51AP1 expression positively related to cancer stemness in 11 cancer types and negatively in THYM and TGCT.

Thirty-seven cancer types were available to perform mRNAsi and mDNAsi scoring analysis in this study. Results showed that the mRNAsi score of 27 cancer types, including stomach adenocarcinoma (STAD), STES, BRCA, LUAD, and lung squamous cell carcinoma (LUSC), were significant positive related to RAD51AP1 expression (Figure 5A). The mDNAsi score of GBM, LGG, SKCM, LUAD, LUSC, BRCA, *etc.* 11 cancer types were also significant positive correlated with RAD51AP1 (Figure 5B). On the contrary, in THYM and testicular germ cell tumors (TGCT), the mDNAsi score significantly negatively correlated with RAD51AP1 (Figure 5B).

### RAD51AP1 alteration might not associate with mutation and be widely involved in RNA modification

**FIGURE 6:**
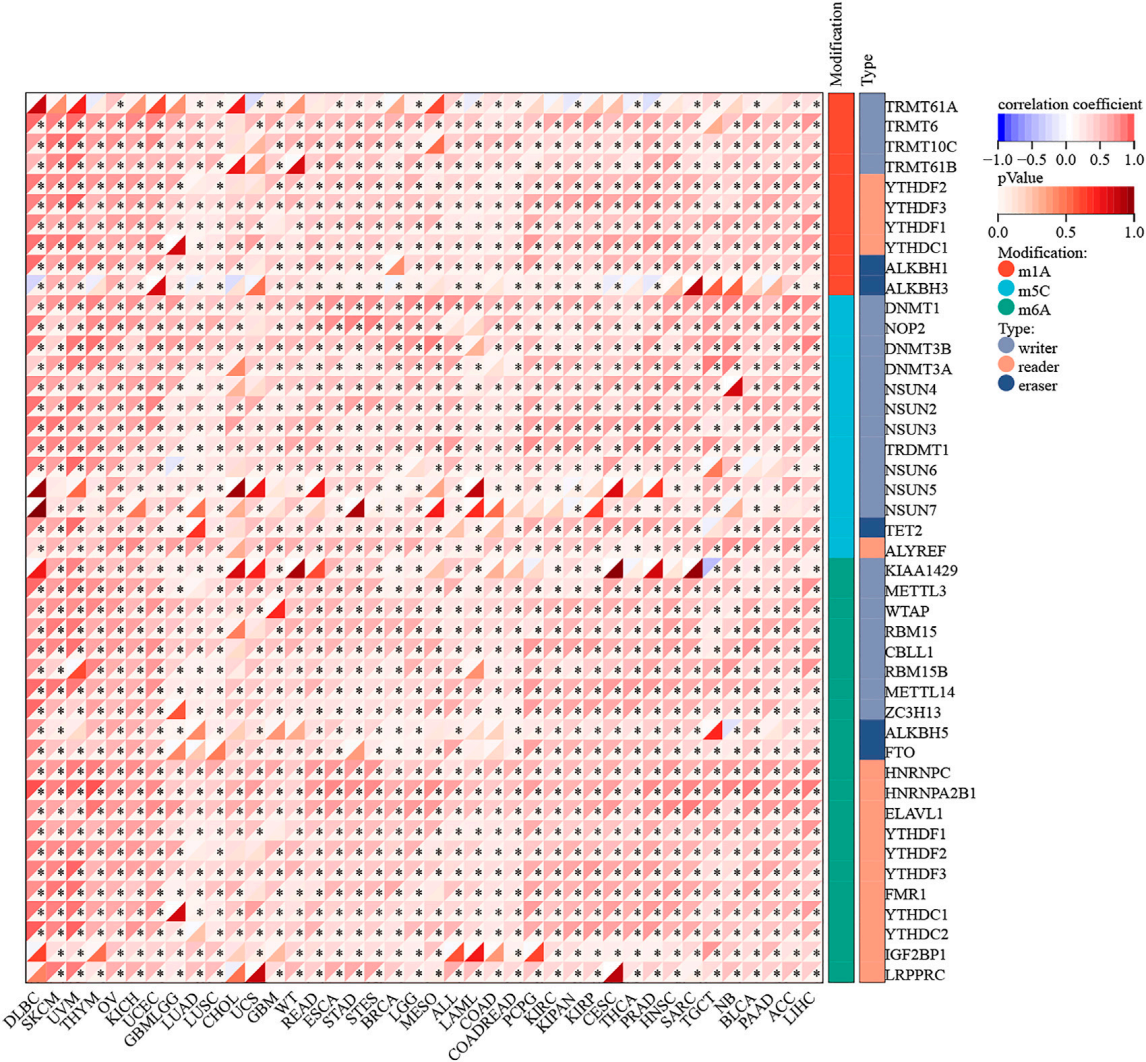
RNA modification correlation analysis of RAD51AP1. RAD51AP1 expression was widely positively associated with 44 RNA modification maker genes (with 10 in m1A, 13 in m5C, and 21 in m6A) expression in most cancers.

The mutation data of RAD51AP1 in the tumor was rare. Only seven cancer types contained available mutation data, and the RAD51AP1 alteration might not associate with its mutation in most of these cancers (Supplementary Figure S4). We further investigated the correlation between RAD51AP1 expression and 44 maker genes of all three types of RNA modification processes (including m1A, m5C, and m6A). In most cancers, the RAD51AP1 expression was usually significant positive related to RNA modification marker gene expression (Figure 6).

## Tumor-associated immune analysis

### Multiple algorithms verified that RAD51AP1 was closely related to tumor immune cell infiltration

**FIGURE 7:**
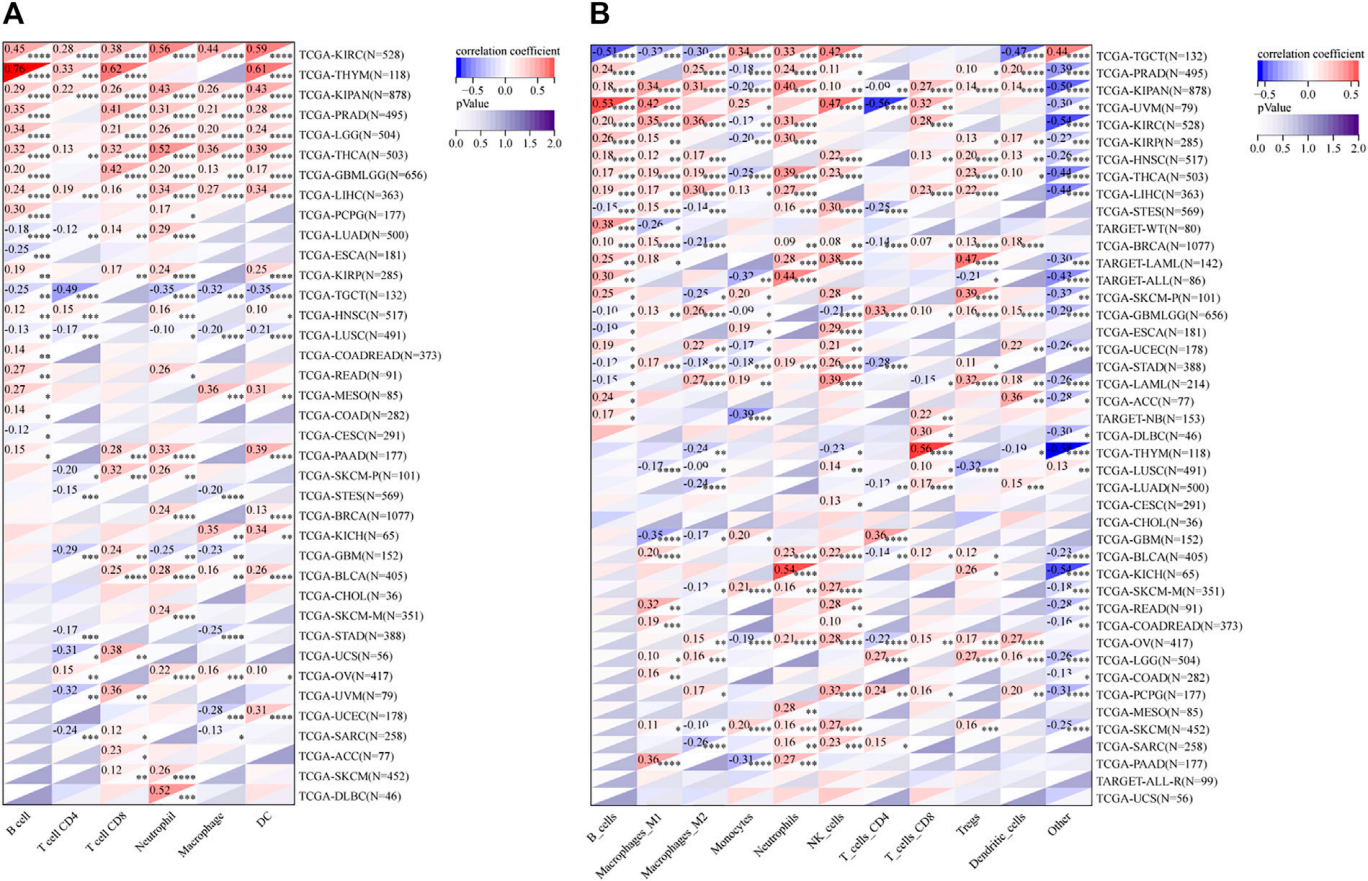
Correlation analysis between RAD51AP1 and tumor immune cell infiltration in TIMER and QUANTISEQ. **(A)** TIMER. The expression of RAD51AP1 was significantly related to tumor immune cell infiltration in 37 cancer types. **(B)** QUANTISEQ. The correlation was significant in 41 cancer types.

The infiltration of B cells, CD4^+^ T cells, CD8^+^ T cells, Neutrophils, Macrophages, and dendritic cells (DCs) in each tumor were analyzed by TIMER. Results showed that 37 of all 38 enrolled cancer types presented a significant correlation between RAD51AP1 expression and tumor immune infiltration levels (Figure 7A). In kidney renal clear cell carcinoma (KIRC), THYM, LGG, THCA, liver hepatocellular carcinoma (LIHC), *etc.* several cancer types, the expression of RAD51AP1 were both positively correlated to CD8^+^ T cells and DCs infiltration. QUANTISEQ algorithm also demonstrated that the expression of RAD51AP1 significantly correlated with immune cell infiltration in multiple cancers (Figure 7B). Highly expressed RAD51AP1 indicated high CD8^+^ T cells, natural killer (NK) cells, and DCs infiltration in head and neck squamous cell carcinoma (HNSC), BRCA, pheochromocytoma and paraganglioma (PCPG), *etc.* and high Macrophages_M2 and Treg cells infiltration in THCA, LIHC, glioma (GBMLGG), *etc.* We further verified the comprehensive correlation between TICs and RAD51AP1 expression in pan-cancer *via* four other independent tumor-immune infiltration level algorithms, including EPIC, MCPcounter, CIBERSORT, and XCELL (Supplementary Figure S5).

### RAD51AP1 correlated with most immune regulatory genes and immune checkpoint genes, including PD-L1 in multiple cancers

**FIGURE 8:**
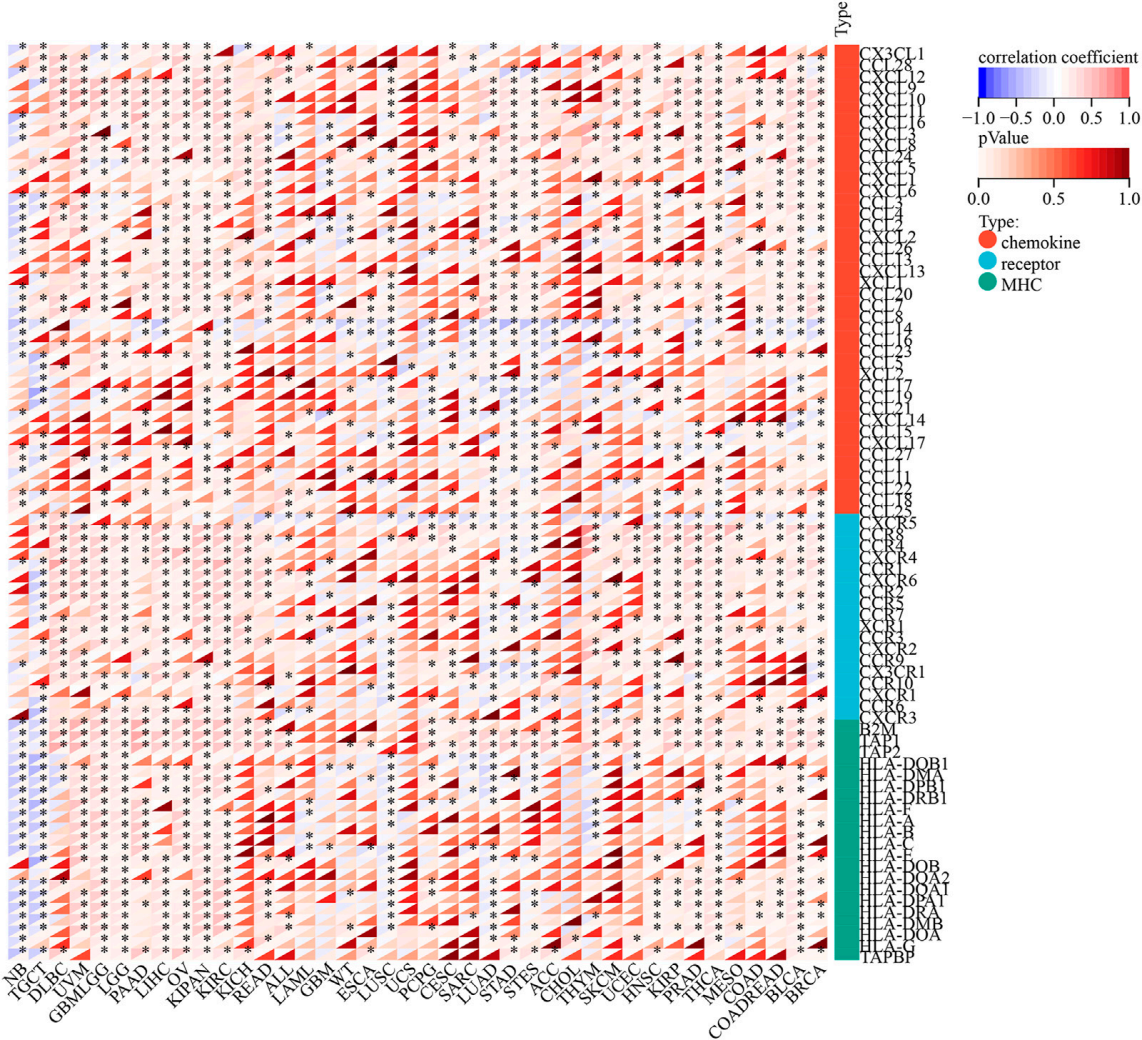
Relationship between RAD51AP1 and chemokine and its receptors genes in pan-cancer. The expression of RAD51AP1 was significantly correlated with CXC and CC family, chemokine receptors, and MHC class I and II in various cancers. MHC, major histocompatibility complex.

**FIGURE 9:**
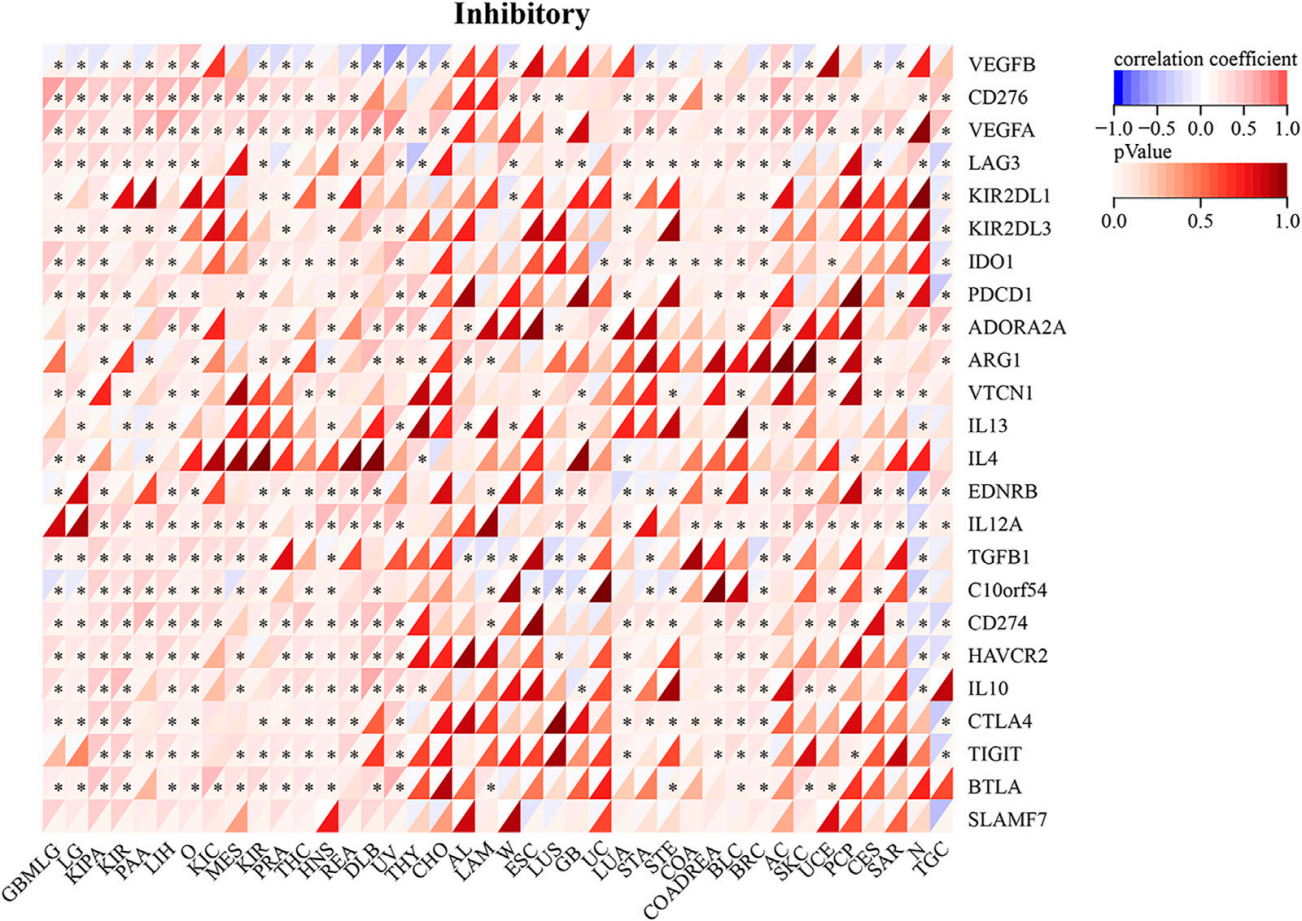
Relationship between RAD51AP1 and immune checkpoint inhibitory genes in pan-cancer. RAD51AP1 presented significant correlations with PDCD1, CD274(PD-L1), CTLA4, *etc.* immune checkpoint inhibitory genes in multiple cancers.

RAD51AP1 expression was significantly correlated with the expression of chemokine and its receptors genes such as CXC and CC family, major histocompatibility complex (MHC) class I and II (Figure 8), immune suppression, and stimulation genes (Supplementary Figure S6) in most cancers. For instance, in LUAD, the expression of RAD51AP1 was positively related to CXCL-5, CXCL-6, CXCL-8, CXCL-9, CXCL-10, *etc.* chemokine genes and negatively related to HLA-DMA, HLA-DMB, *etc.* MHC genes. We also found that the expression of immune checkpoint inhibitory (Figure 9) and stimulatory (Supplementary Figure S7) genes were significantly correlated with RAD51AP1 in various cancer types. Twenty-six types of cancer with highly expressed RAD51AP1 presented high CD274 (PD-L1) expression, including LGG, KIRC, and PAAD.

### RAD51AP1 usually negatively correlated with IPS and ESTIMATE scores in cancers

**FIGURE 10:**
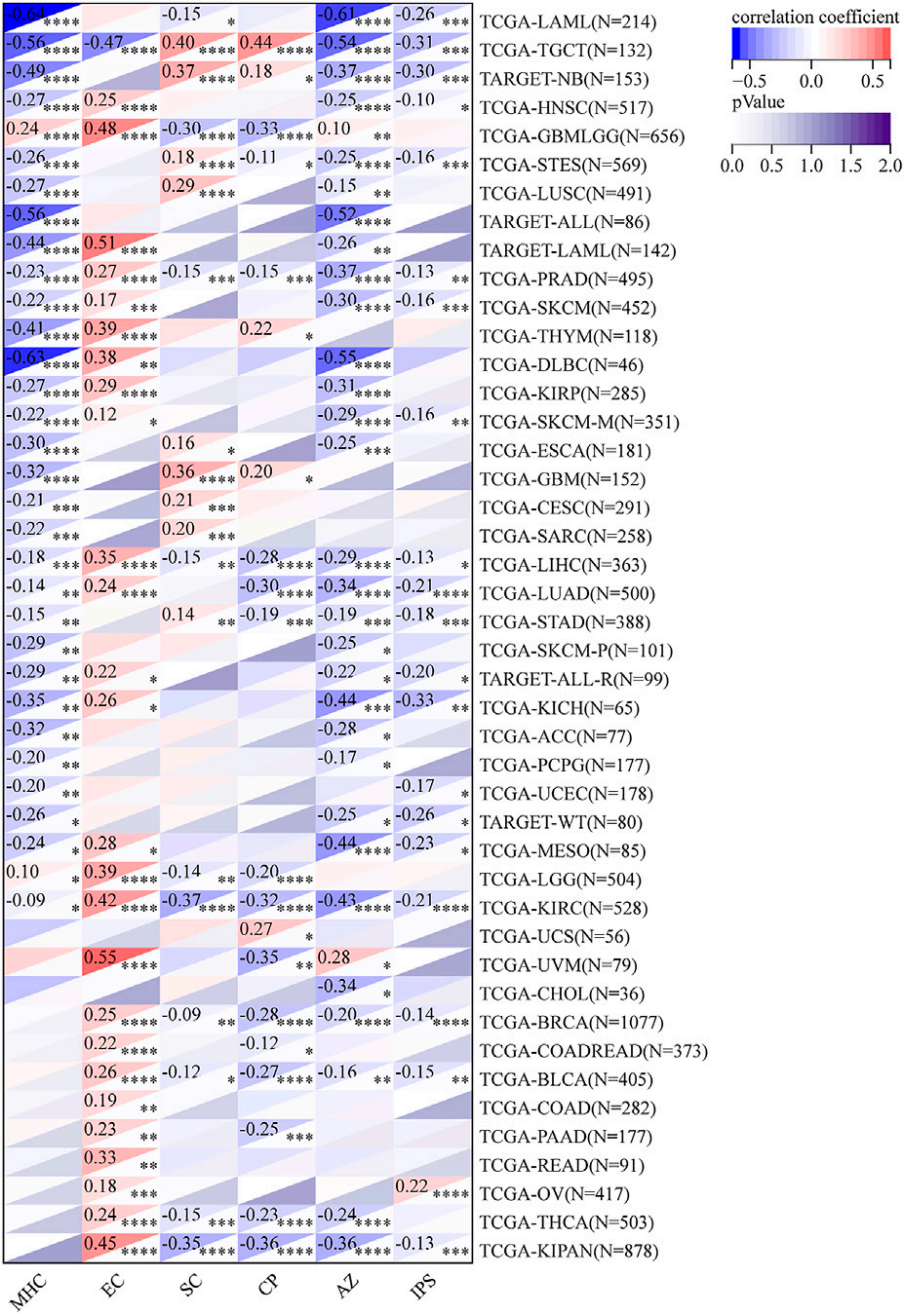
Comprehensive assessment of RAD51AP1 in tumor immune *via* IPS. In most cancers except ovarian serous cystadenocarcinoma (OV), RAD51AP1 expression negatively correlated with immunophenoscore. IPS, immunophenoscore; MHC, antigen processing; EC, effector cells; SC, suppressor cells; CP, checkpoints.

Comprehensive immune infiltration assessments were analyzed *via* IPS and ESTIMATE in R software. Results showed that RAD51AP1 expression was significantly negatively correlated with IPS score in most cancers, including kidney chromophobe (KICH), LAML, and LUAD (Figure 10). The opposite result was only observed in ovarian serous cystadenocarcinoma (OV). The ESTIMATE algorithm also demonstrated similar correlations in multiple cancers (Supplementary Figure S8).

### RAD51AP1 was significantly associated with MSI, TMB, and tumor heterogeneity

**FIGURE 11:**
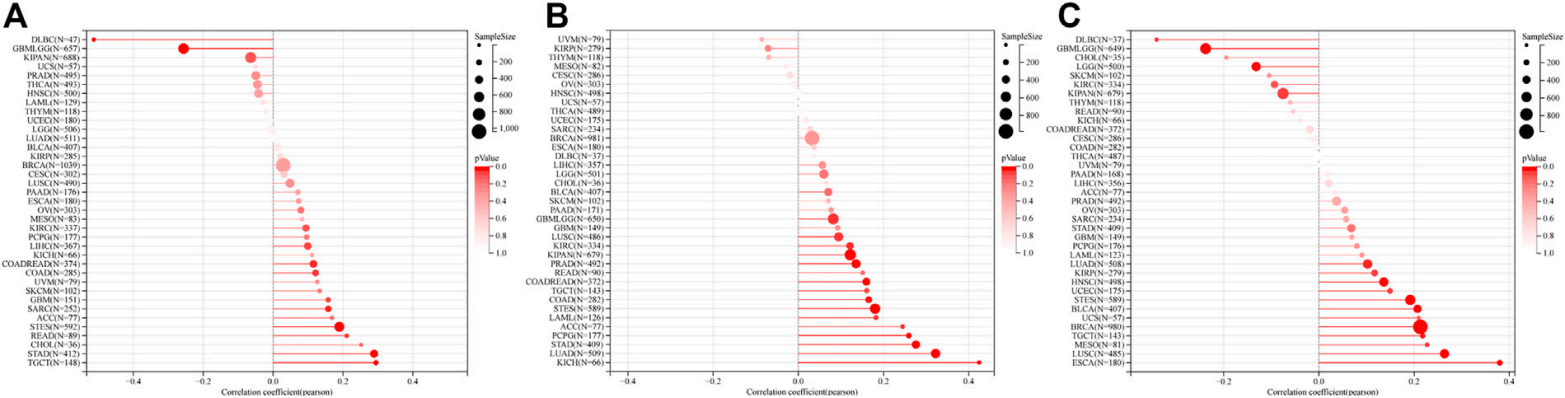
MSI, TMB, and tumor heterogeneity correlation analysis of RAD51AP1 in pan-cancer. **(A)** MSI. Positively correlated in COAD (*p =* 0.041), COADREAD (*p =* 0.026), STES (*p* < 0.001), SARC (*p =* 0.012), STAD (*p* < 0.001), READ (*p =* 0.048) and TGCT (*p* < 0.001). Negatively correlated in GBMLGG (*p* < 0.001) and DLBC (*p* < 0.001). **(B)** TMB. Positively correlated in 14 cancer types including GBMLGG (*p =* 0.037), LUAD (*p* < 0.001), COAD (*p =* 0.005), COADREAD (*p =* 0.002), LAML (*p =* 0.044), STES (*p* < 0.001), KIPAN (*p =* 0.001), STAD (*p* < 0.001), PRAD (*p =* 0.003), KIRC (*p =* 0.027), LUSC (*p =* 0.037), PCPG (*p* < 0.001), ACC (*p =* 0.032) and KICH (*p* < 0.001). **(C)** MATH. Positively correlated in LUAD (*p =* 0.021), BRCA (*p* < 0.001), ESCA(*p* < 0.001), STES (*p* < 0.001), UCEC (*p =* 0.049), HNSC(*p =* 0.002), LUSC(*p* < 0.001), MESO(*p =* 0.041), TGCT (*p =* 0.009) and BLCA (*p* < 0.001). Negatively correlated in GBMLGG (*p* < 0.001), LGG (*p =* 0.003), KIPAN (*p =* 0.049) and DLBC (*p =* 0.039).

The expression of RAD51AP1 was significantly positively correlated with MSI in COAD, COADREAD, STES, SARC, STAD, READ, and TGCT, and negatively in GBMLGG and lymphoid neoplasm diffuse large B-cell lymphoma (DLBC) (Figure 11A). TMB was positively related to RAD51AP1 in 14 cancer types, including GBMLGG, LUAD, and COAD. (Figure 11B). RAD51AP1 also positively correlated with MATH in 10 cancer types, including LUAD, BRCA, and ESCA, and negatively in LGG, KIPAN, and DLBC (Figure 11C).

### RAD51AP1 was up-regulated in various cancer cell lines and promoted cancer cells proliferation

**FIGURE 12:**
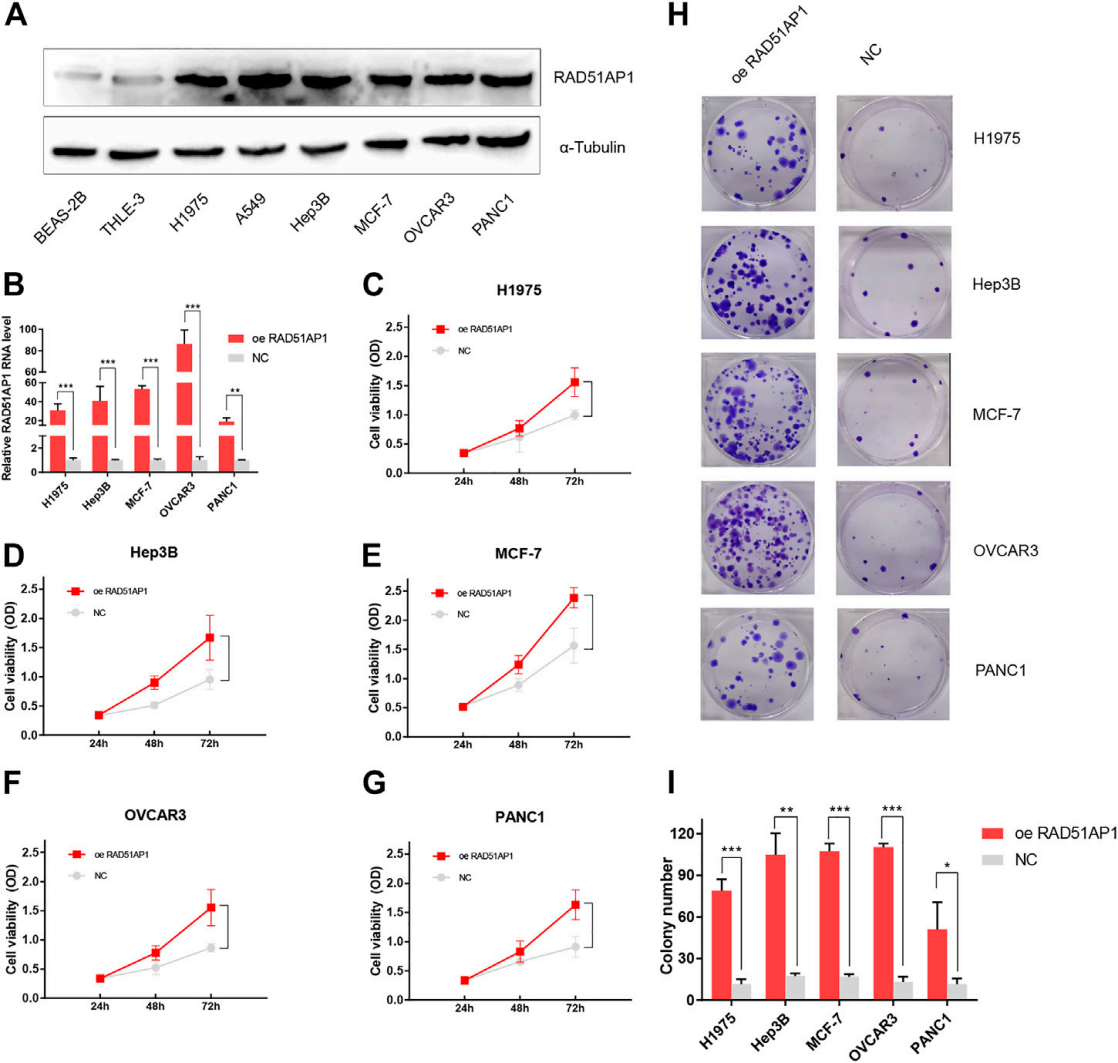
Validation of the oncogenic role of RAD51AP1 by vitro experiment. The protein expression level of RAD51AP1 in each cell line was tested by western blot. RAD51AP1 was up-regulated in various cancer cells, including H1975, A549, Hep3B, MCF-7, OVCAR3, and PANC1, compared to normal cells BEAS-2B and THLE-3 **(A)**. Then, we selected H1975, Hep3B, MCF-7, OVCAR3, and PANC1 to represent each type of cancer for further validation. After successfully transfecting vector or RAD51AP1 OE plasmids in each cancer cell line, verified *via* qPCR **(B)**, we performed CCK8 and colony formation assays. Results showed that RAD51AP1 significantly enhanced cancer cells viability **(C–G)** and promoted cancer cells proliferation **(H,I)**.

To further authenticate the oncogenic role of RAD51AP1 in cancers, we tested the protein expression level of RAD51AP1 in each cell line by western blot. The expression of RAD51AP1 in lung cancer cell lines H1975 and A549, hepatocellular carcinoma cell line Hep3B, breast cancer cell line MCF-7, ovarian cancer cell line OVCAR3, and pancreatic cancer cell line PANC1 were higher than normal lung epithelial cell line BEAS-2B and liver epithelial cell line THLE-3 (Figure 12A). We further transfected vector or RAD51AP1 OE plasmids in H1975, Hep3B, MCF-7, OVCAR3, and PANC1. The transfection effectiveness in each cell line was tested by qPCR (Figure 12B). Using the CCK8 kit, we found that upregulation of RAD51AP1 significantly improved cell viability in all five cancer cell lines (Figures 12C–G). Moreover, the colony formation assays also demonstrated that RAD51AP1 significantly promoted the proliferation of each cancer cell line (Figures 12H,I).

## Discussion

RAD51AP1, also named PIR51, was first discovered in 1997 (Kovalenko et al., 1997; Mizuta et al., 1997). It can stimulate the D-loop formation by binding RAD51 and single-end invasion intermediate (SEI) together to fulfill the HR process in the normal cell (Modesti et al., 2007). Unlike the general genome stability function of RAD51AP1, it also presents oncogene-like functions in breast, ovarian, and lung cancers as highly expressed in tumor tissues and correlated with poor prognosis (Sankaranarayanan et al., 2015; Pathania et al., 2016; Chudasama et al., 2018; Wu et al., 2019; Bridges et al., 2020; Bridges et al., 2021). In line with these studies, we used the bioinformatics methods to demonstrate that RAD51AP1 was significantly up-regulated in 33 tumor tissues (Figure 2). We also verified that highly expressed RAD51AP1 indicated poor OS and PFS (Figure 3; Supplementary Figures S2,S3) in most tumors. We further demonstrated that RAD51AP1 was up-regulated in typical cancer cell lines, and overexpressed RAD51AP1 promoted cancer cell proliferation *in vitro* (Figure 12). These results might indicate that RAD51AP1 plays an oncogene-like role in cancers.

The mechanism of RAD51AP1 in cancer development is still unclear. Using a core stemness algorithm developed by TM Malta et al. (2018), we demonstrated that the expression of RAD51AP1 was positively correlated with mRNAsi score in 27 cancer types and mDNAsi score in 11 cancer types (Figure 5). According to these results, we speculate that RAD51AP1 might promote cancer development by maintaining cancer stemness in multiple cancers. Two studies have demonstrated this mechanism in breast cancer and CRC (Bridges et al., 2020; Bridges et al., 2021). They found that cancer stem cells (CSCs) specific marker genes were significantly down-expressed, and the proportion of CSCs was significantly reduced in RAD51AP1 knock-down cancer cells. In line with these studies, we found that mRNAsi in COAD and both mRNAsi and mDNAsi in BRCA significantly correlated with RAD51AP1 expression (Figure 5). The mDNAsi in COAD also presented a positive correlation tendency, although without statistically significant (Figure 5B). However, the correlation between RAD51AP1 and cancer stemness in other cancers has not been reported yet. Although our study demonstrated these positive correlations in bioinformatics levels, more vitro and vivo experiments-based evidence should be found to verify this hypothesis in the future. Meanwhile, we also observed the opposite results that the mDNAsi of THYM and TGCT were negatively correlated to RAD51AP1 expression (Figure 5B). The THYM originates from thymic epithelial cells and has distinct clinical features and gene expression profiles compared to other cancers (Oberndorfer and Mullauer, 2020). For instance, nearly one-third of THYM patients have comorbid autoimmune diseases, including myasthenia gravis, pure red cell aplasia, and hypogammaglobulinemia (Girard et al., 2015). Studies also showed that THYM presented extremely lower TMB and higher GTF21 p. (Leu404His) point mutations incidence (Petrini et al., 2014; Radovich et al., 2018). For TGCT, a whole-exome sequencing (WES) analysis of 42 cases also presented significantly lower mutation probability and a completely different mutation spectrum (Litchfield et al., 2015). These investigations suggested that both THYM and TGCT might have different mechanisms of tumorigenesis and might explain these opposite results.

On the other hand, RNA modification also plays a widespread role in tumor proliferation, invasion, metastasis, and immune regulation (Barbieri and Kouzarides, 2020). The effect of RAD51AP1 on RNA modification in tumors has not been reported yet. Our study found that the expression of RAD51AP1 was significantly positively correlated with the expression of m1A, m5C, and m6A marker genes in various tumors (Figure 6). Under certain assumptions, we speculate that RAD51AP1 might also affect tumor cells’ malignant phenotype *via* RNA modification regulation.

Another novel finding was that RAD51AP1 expression was significantly correlated to TICs levels in multiple cancers. The TICs can regulate tumor proliferation, metastasis, and drug resistance by affecting tumor-related immune processes (Galli et al., 2020). TICs are functionally divided into two categories: 1) tumor cell growth inhibition including CD8^+^ T, Th1 CD4^+^ T, Th9 CD4^+^ T, plasma, memory B, NK cells, and DCs; 2) tumor cell growth or immune escape stimulation including Treg, Breg, macrophage M2, and myeloid-derived suppressor cells, (MDSCs) (Kuang et al., 2009; Brandau et al., 2011; Shi et al., 2013; Kim and Cantor, 2014; Liu and Cao, 2016; Mao et al., 2017; Rivera Vargas et al., 2017; Kobayashi et al., 2019; Xie et al., 2021). We analyzed the specific correlation between RAD51AP1 expression and each cell mentioned above in pan-cancer *via* six independent algorithms. Results showed that RAD51AP1 was broadly correlated to TICs in various cancers in a diverse manner (Figure 7; Supplementary Figure S5). At this stage of understanding, we believe that RAD51AP1 may have an essential role in the tumor microenvironment (TME) and may become a candidate indicator to distinguish between so-called “hot” or “cold” tumors.

We further verified that RAD51AP1 expression was significantly correlated with the expression of MHC, chemokines and their receptors, and immune checkpoint inhibitory and stimulatory genes in various tumors (Figure 8, Figure 9; Supplementary Figures S6,S7). The RAD51AP1 expression was also broadly negatively correlated to IPS and ESTIMATE scores (Figure 10; Supplementary Figure S8). IPS is a comprehensive TICs scoring algorithm developed by *P.* Charoentong et al. (2017). Four aspects: effector cells infiltration, immunosuppressive cells infiltration, checkpoint gene expression, and antigen processing gene expression, were synthetically calculated in this method. A higher IPS score often indicates a better prognosis in cancer patients. Our results showed that RAD51AP1 was significantly negatively correlated with IPS score in multiple cancers, including KICH, LAML, and LUAD, indicating a negative correlation with prognosis among these cancer types (Figure 10). These results were basically in accordance with the survival analysis results mentioned above (Figure 3). The higher ESTIMATE scores, developed by *K.* Yoshihara et al. (2013) also indicate a better prognosis in cancers (Liu et al., 2018b). Our study also found significant negative correlations between RAD51AP1 expression and ESTIMATE scores in various cancers (Supplementary Figure S8). Therefore, RAD51AP1 may be widely involved in cancer immune response and broadly participate in TME regulations, leading to a worse prognosis. However, the specific effects of RAD51AP1 on TME and whether these effects will or will not lead to cancer development in multiple cancers still need to be explored in the future.

RAD51AP1 might also be positively correlated to tumor heterogeneity in multiple cancers. MATH is a standard algorithm in tumor heterogeneity assessment. The higher MATH often indicated higher tumor heterogeneity and worse prognosis in cancers (Mroz and Rocco, 2013; Ma et al., 2017; Rajput et al., 2017). Our results showed that RAD51AP1 was positively correlated with MATH score in 10 cancer types (Figure 11C). RAD51AP1 itself is a crucial protein in HR, as mentioned above. When DNA double-strand break (DSB) occurs in a normal cell, it can recruit RAD51, bind to broken single-stranded DNA (ssDNA) to form presynaptic filaments, and form D-loop to fulfill DNA repair (Richardson, 2005; Moynahan and Jasin, 2010). Thus we speculate that the genome homeostasis-maintaining function of RAD51AP1 may also improve tumor heterogeneity. This hypothesis may also explain another finding in our study that RAD51AP1 expression was positively correlated with TMB in various tumors (Figure 11B). Moreover, TMB and MATH are effective biomarkers of immune checkpoint inhibitors (ICIs) treatment, and their increment in the tumor may indicate better ICIs therapy efficacy (Gao et al., 2020; Jiang et al., 2021). Interestingly, we also found that cancer with higher RAD51AP1 expression often presented higher MSI (Figure 11A), which is positively correlated to ICIs therapy efficacy in CRC and other cancers (Dudley et al., 2016). Meanwhile, RAD51AP1 was also usually positively correlated with the expression of PD-L1 (Figure 9), another positive predictor of PD-1/PD-L1 inhibitors (Yang et al., 2021). Therefore, from this standpoint, we presume that RAD51AP1 may become a potential biomarker candidate in ICIs treatment in pan-cancers.

However, there are several limitations to this study. First, in the mutation correlation analysis of RAD51AP1, the sample size of mutated RAD51AP1 was far less than the wild-type group. Although one of the differences was statistically significant, the extremely asymmetric sample size in the different groups may still lead to an inaccuracy result. Second, as the staging and therapeutic approach data were insufficient, we could hardly further investigate the detailed effects of RAD51AP1 in each cancer stage or in any anti-cancer treatments. Meanwhile, the lack of data in the tumor microenvironment also restrained further validation of the RAD51AP1 effect on tumor-associated immune at a multi-faceted level. Finally, although we validated the oncogene-like role of RAD51AP1 in several cancers *in vitro* experiments, the underlying mechanism, including signaling pathway regulating, stemness maintaining, RNA modification, and TME regulating, were still only predicted at the bioinformatics level. Further research should focus on the specific mechanism of RAD51AP1 effects on individual cancers.

In conclusion, our study found that RAD51AP1 was highly expressed in most tumors and usually indicated a poor prognosis. The role of RAD51AP1 in enhancing tumor stemness, regulating RNA modification, and affecting the tumor immune microenvironment might compose its carcinogenesis mechanism. It was also usually positively correlated to PD-L1, tumor heterogeneity, TMB, and MSI, suggesting that RAD51AP1 may become a potential predictive biomarker for ICIs therapy. This pilot study might provide several directions for future research.

## Data Availability

The original contributions presented in the study are included in the article/Supplementary Material, further inquiries can be directed to the corresponding authors.
