# Supplementary material for: Systematic pan-cancer analysis showed that RAD51AP1 was associated with immune microenvironment, tumor stemness, and prognosis
Source: Front Genet. 2022 Nov 16;13:971033. doi: 10.3389/fgene.2022.971033 (PMC9708706; doi:10.3389/fgene.2022.971033)
Supplement: Supplementary file 1 [file DataSheet1.DOCX]

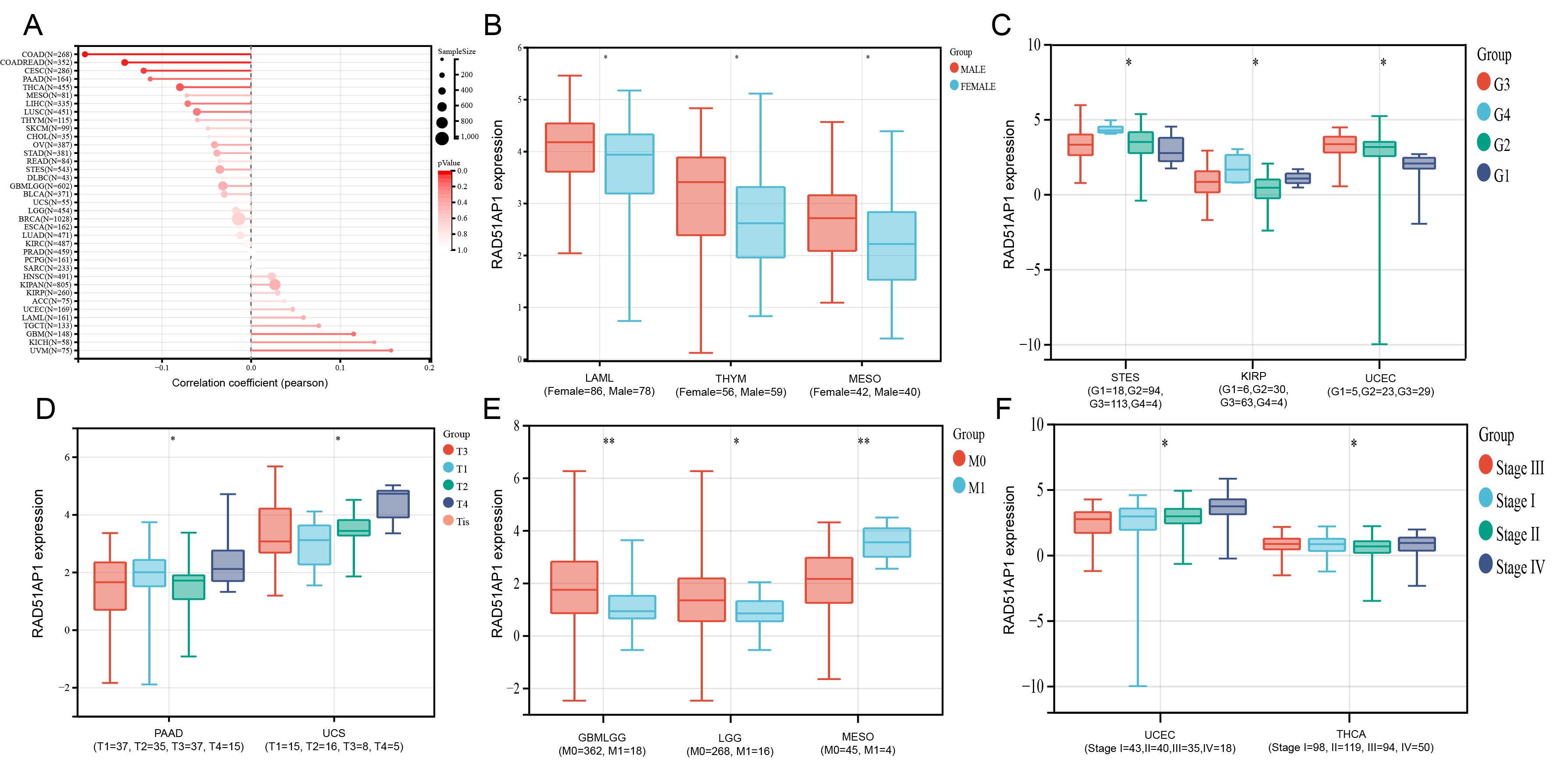


**Supplementary Figure 1 Clinical features related to RAD51AP1 expression**

The high expression of RAD51AP1 was related to younger patients in CESC (*p*=0.042), COAD (*p*=0.002) and COADREAD (*p*=0.008) **(A)**; male in LAML (*p*=0.02), THYM (*p*=0.04) and MESO (*p*=0.05) **(B)**; high-grade differentiation in STES (*p*=0.02), KIRP (*p*=0.04) and UCEC (*p*=0.02) **(C)**; high T stage in PAAD (*p*=0.04) and UCS (*p*=0.03) **(D)**; high M stage in GBMLGG (*p*=8.5e-3), LGG (*p*=0.05) and MESO (*p*=9.2e-3) **(E)**; high TNM stage in UCEC(*p*=0.01) and THCA(*p*=0.03) **(F)**.

**Supplementary Figure 2 Overall survival curves with positive results of RAD51AP expression in pan-cancer**


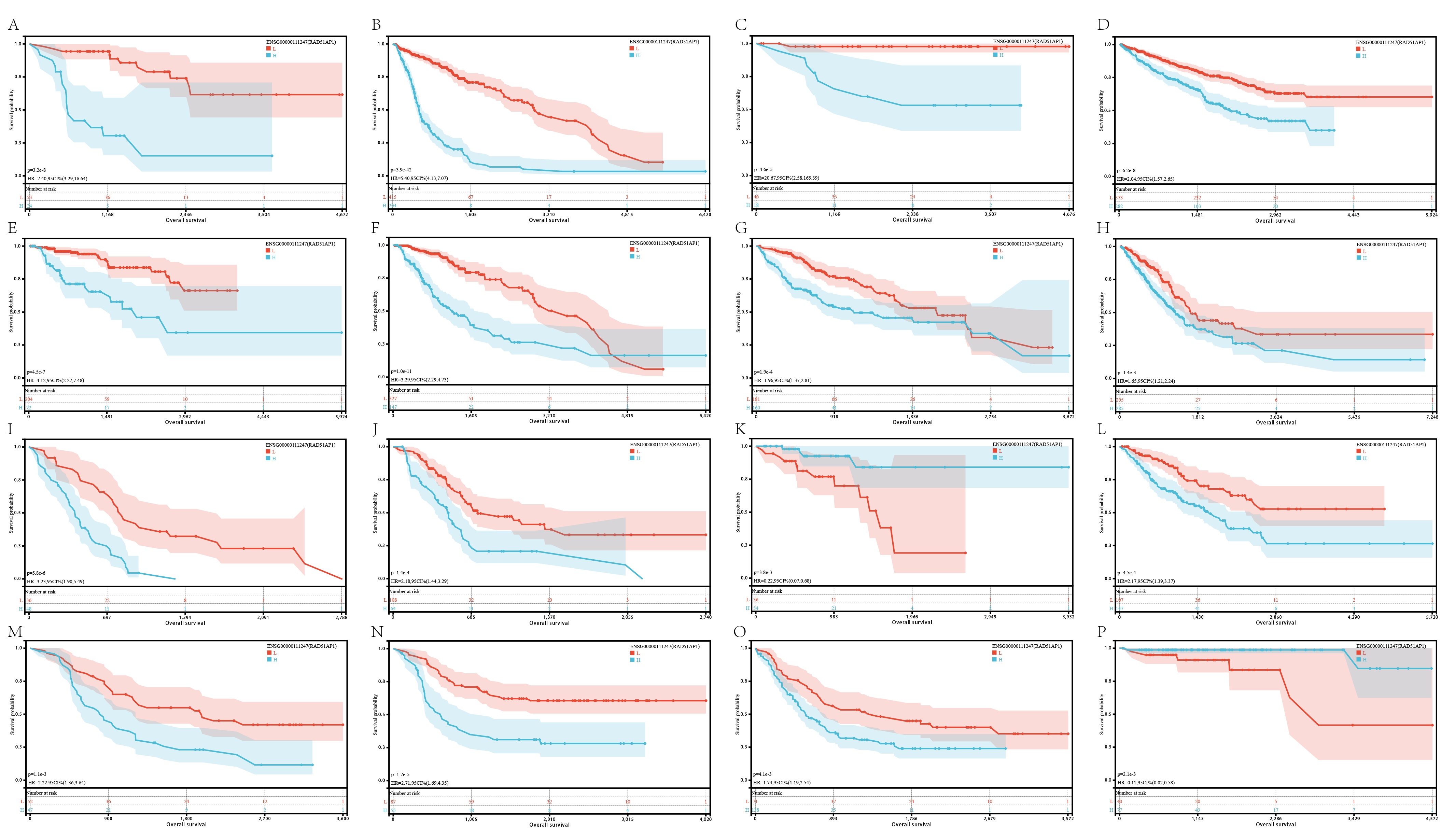


Highly expressed RAD51AP1 linked to poor prognosis in **(A)** ACC, **(B)** GBMLGG, **(C)** KICH, **(D)** KIPAN, **(E)** KIRP, **(F)** LGG, **(G)** LIHC, **(H)** LUAD, **(I)** MESO, **(J)** PAAD, **(L)** SARC, **(M)** TARGET-ALL, **(N)** TARGET-LAML and **(O)** TCGA-LAML, and good prognosis in **(K)** READ and **(P)** THYM. The cut-off value of RAD51AP1 expression was calculated via MaxStat in R software. L, low expression; H, high expression.

**Supplementary Figure 3 Progression-free survival curves with positive results of RAD51AP expression in pan-cancer**


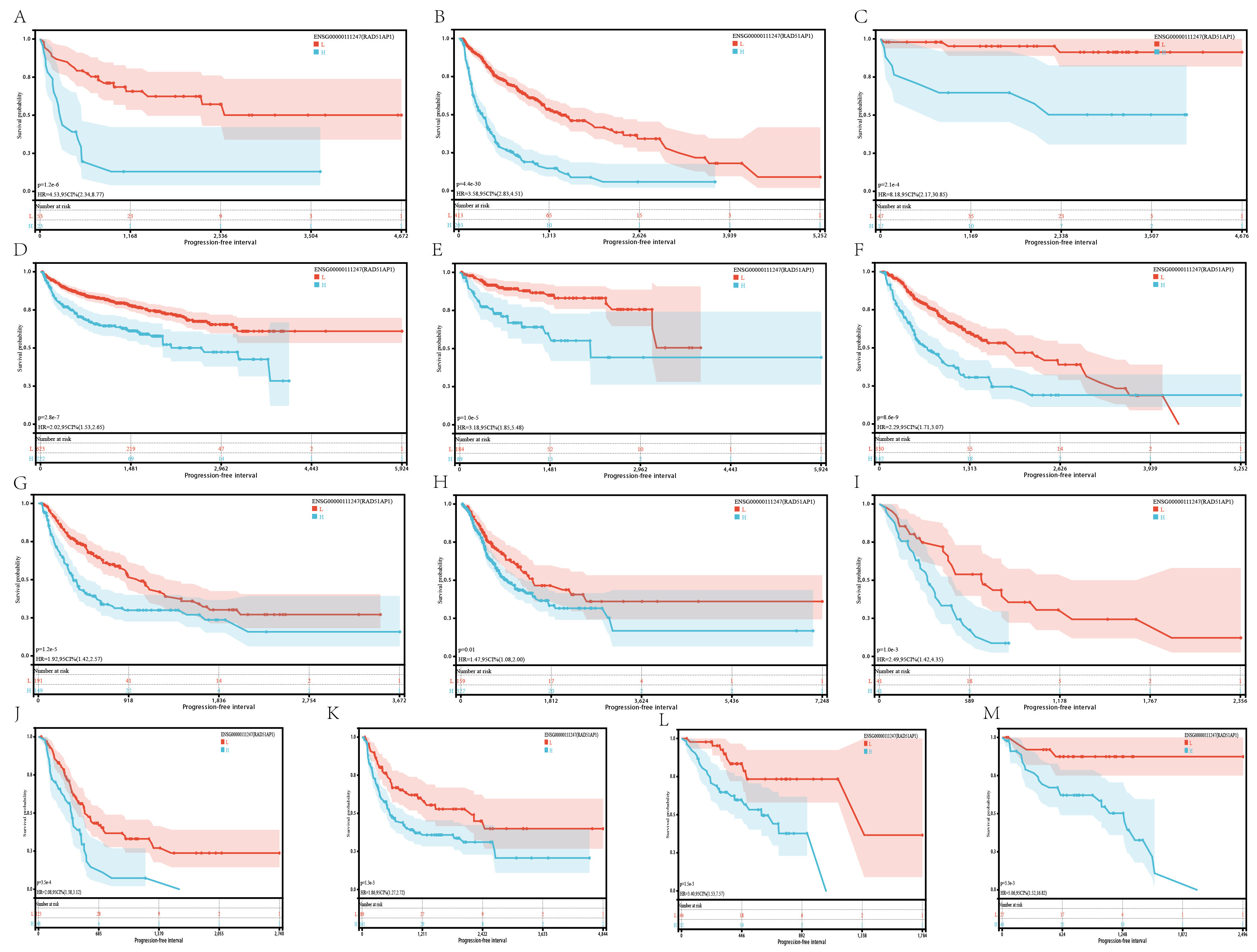


Highly expressed RAD51AP1 linked to poor prognosis in **(A)** ACC, **(B)** GBMLGG, **(C)** KICH, **(D)** KIPAN, **(E)** KIRP, **(F)** LGG, **(G)** LIHC, **(H)** LUAD, **(I)** MESO, **(J)** PAAD, **(K)** SARC, **(L)** SKCM-P and **(M)** UVM. The cut-off value of RAD51AP1 expression was calculated via MaxStat in R software. L, low expression; H, high expression.


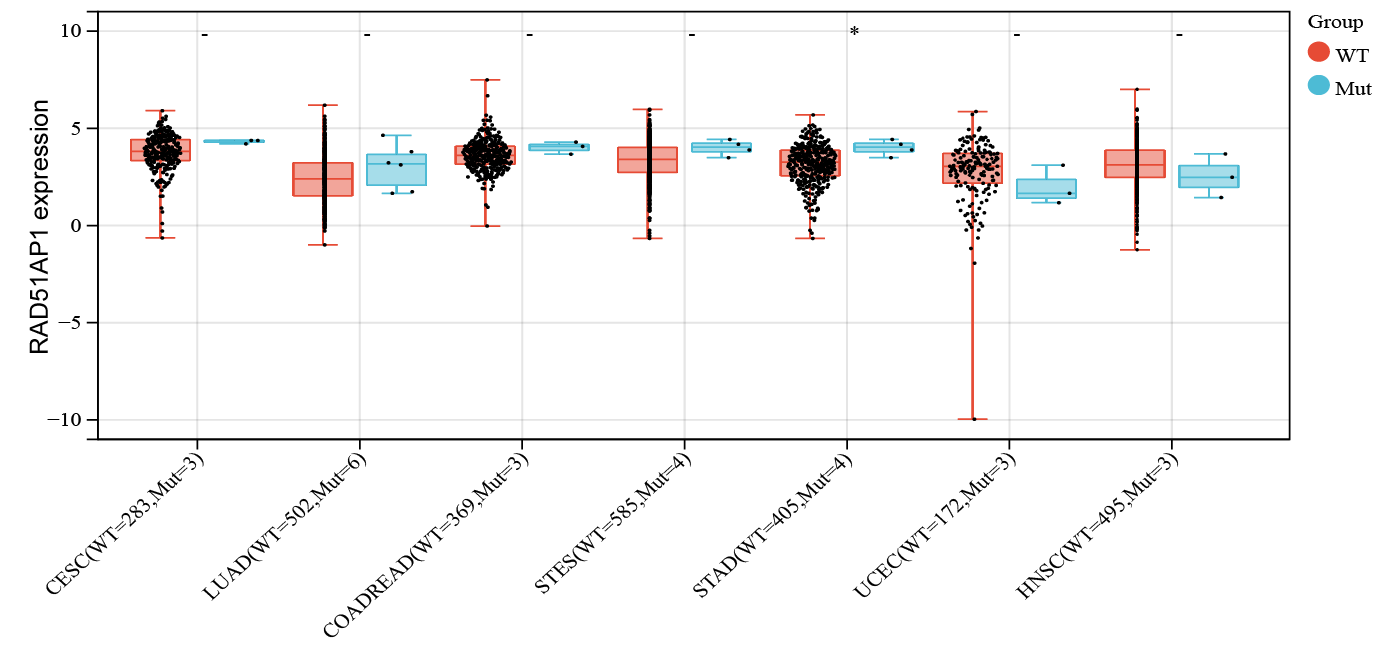


**Supplementary Figure 4 Gene mutation correlation analysis of RAD51AP1**

The RAD51AP1 mutation in cancers is rare, and the RAD51AP1 alteration might not associate with its mutation in most cancers. WT, wild type; Mut, mutation.

**Supplementary Figure 5 Correlation analysis between TICs and RAD51AP1 expression in other four independent algorithms**


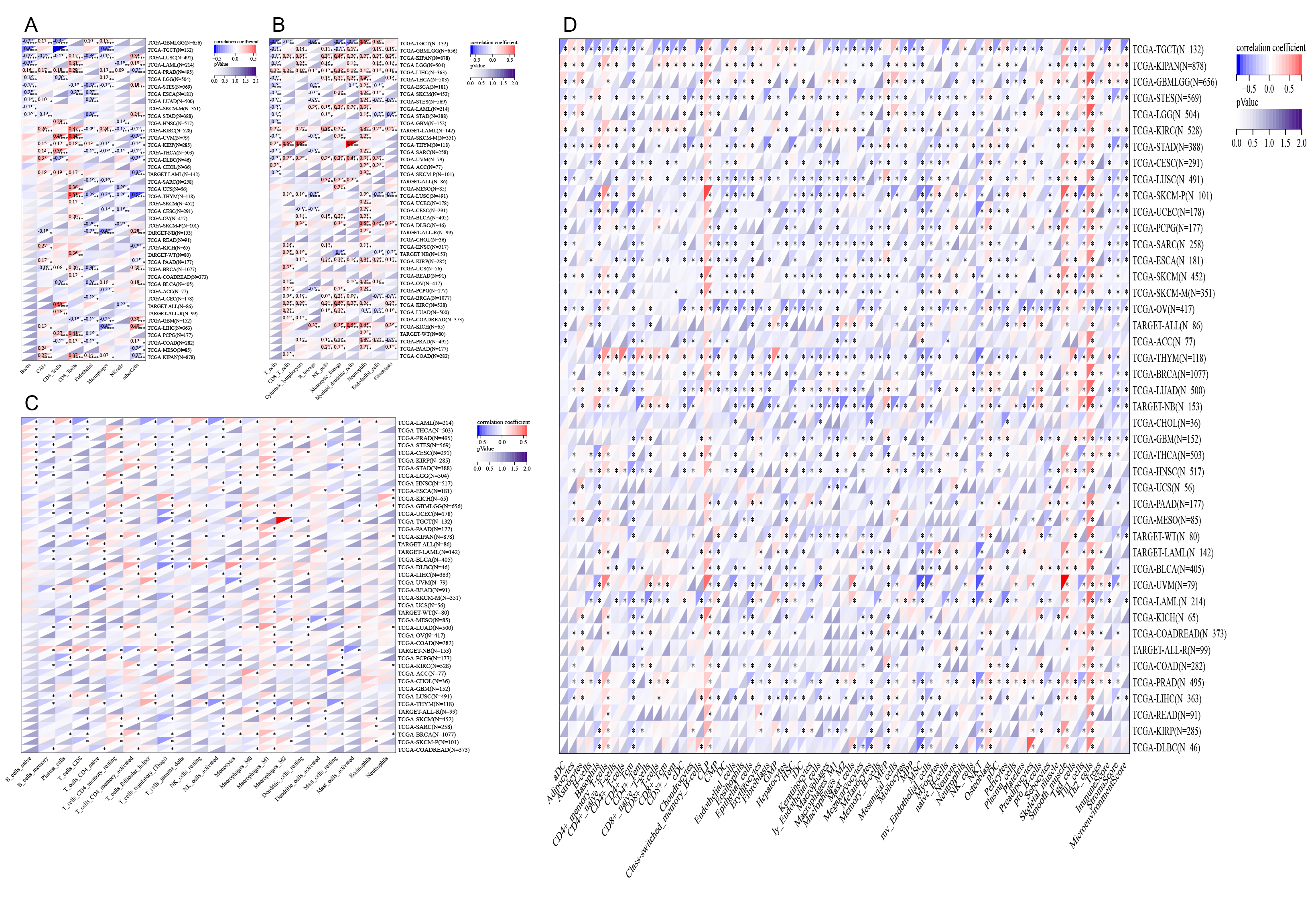


Multiple cancers presented a significant correlation between RAD51AP1 expression and tumor immune cell infiltration according to **(A)** EPIC**, (B)** MCPcounter**, (C)** CIBERSORT, and **(D)** XCELL analysis, respectively.


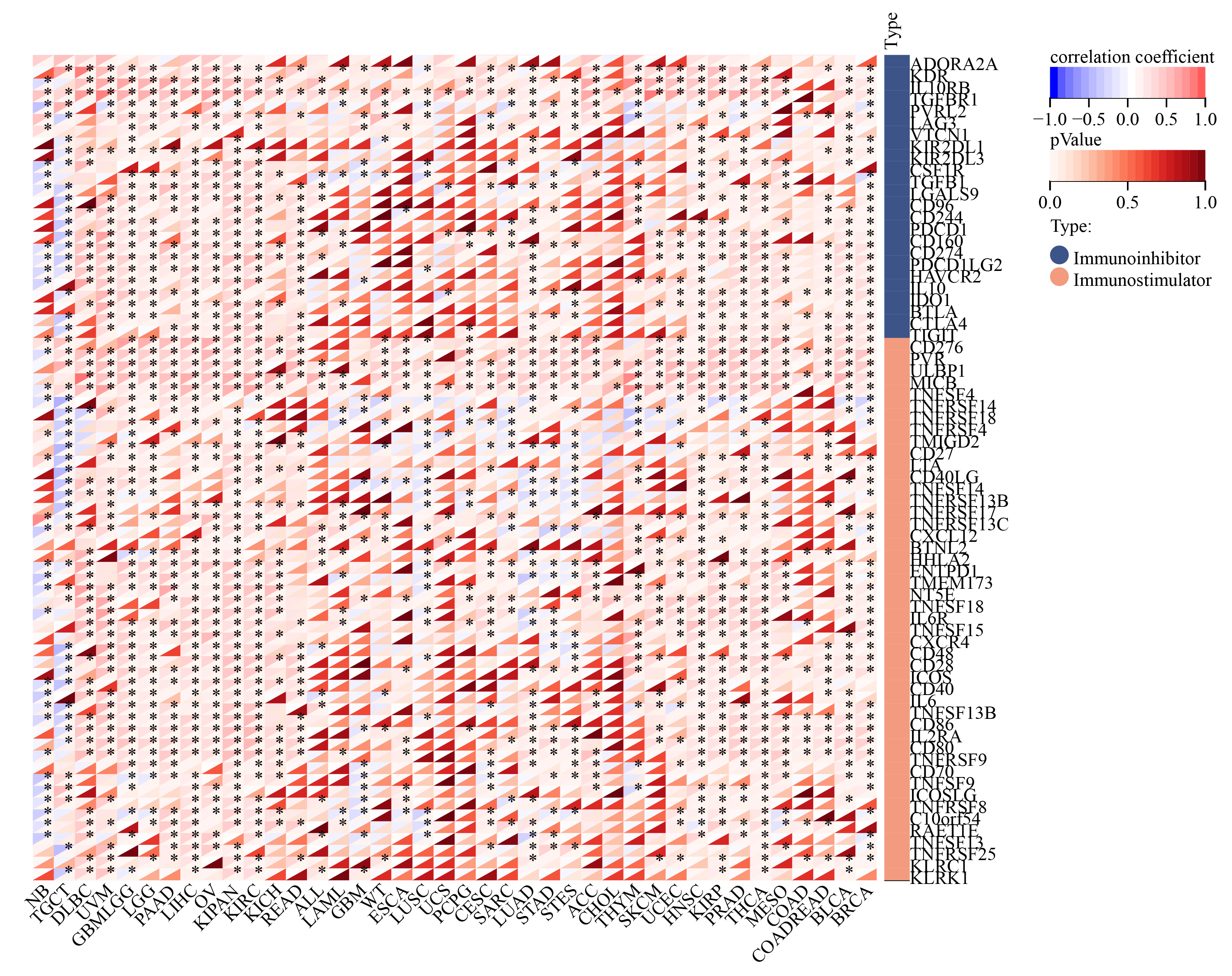


**Supplementary Figure 6 Relationship between RAD51AP1 and immunoinhibitor or immunostimulator genes in pan-cancer**

The expression of RAD51AP1 was significantly correlated with PDCD1, CTLA4, etc. immunoihbibitor genes and with CXCR4, CD40, etc. immunostimulator genes in various cancers.


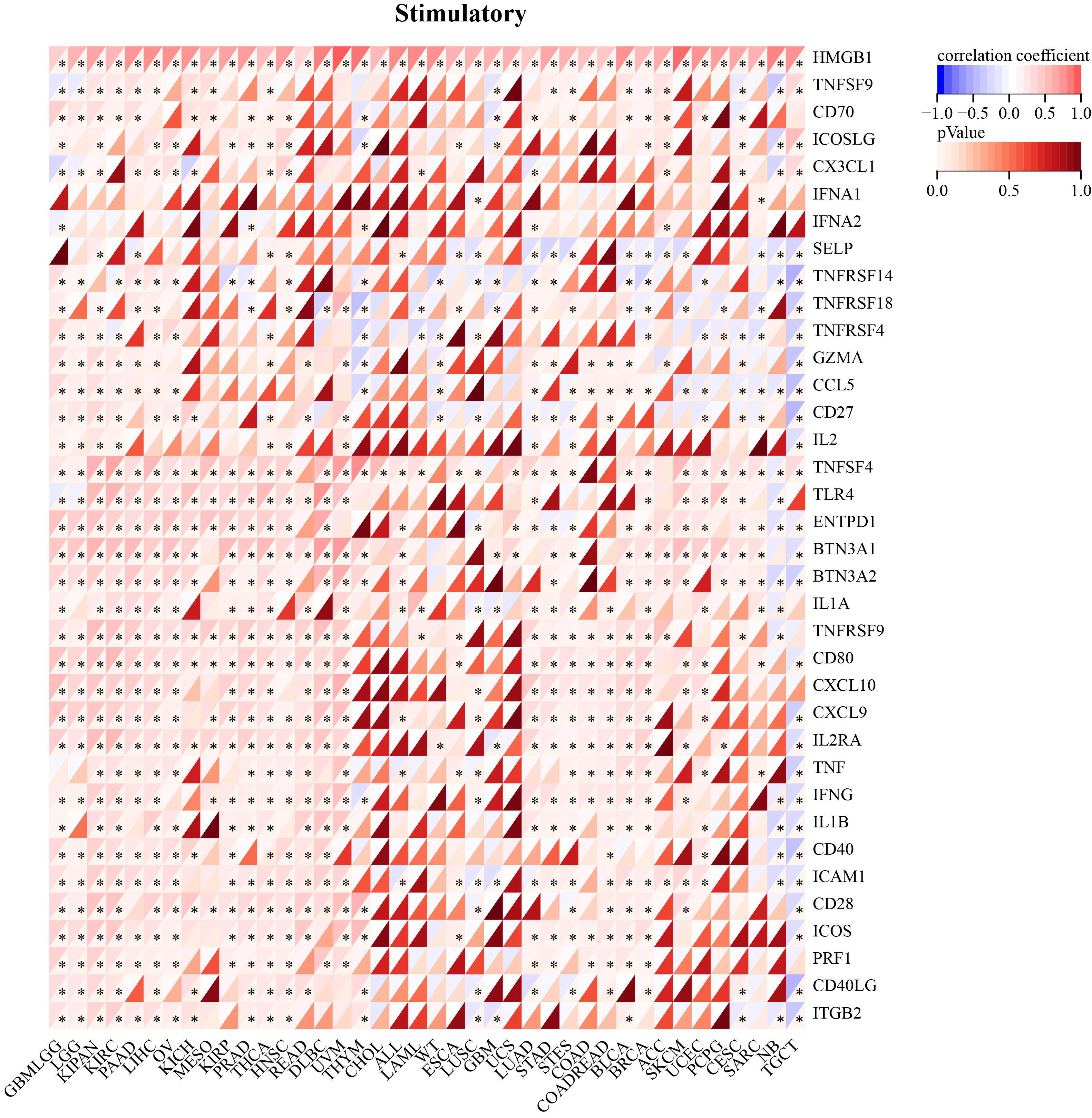


**Supplementary Figure 7 Relationship between RAD51AP1 and immune checkpoint stimulatory genes in pan-cancer**

RAD51AP1 presented significant correlations with HMGB1, CD70, etc. immune checkpoint stimulatory genes in multiple cancers.

**Supplementary Figure 8 Comprehensive assessment of RAD51AP1 in tumor immune via ESTIMATE**


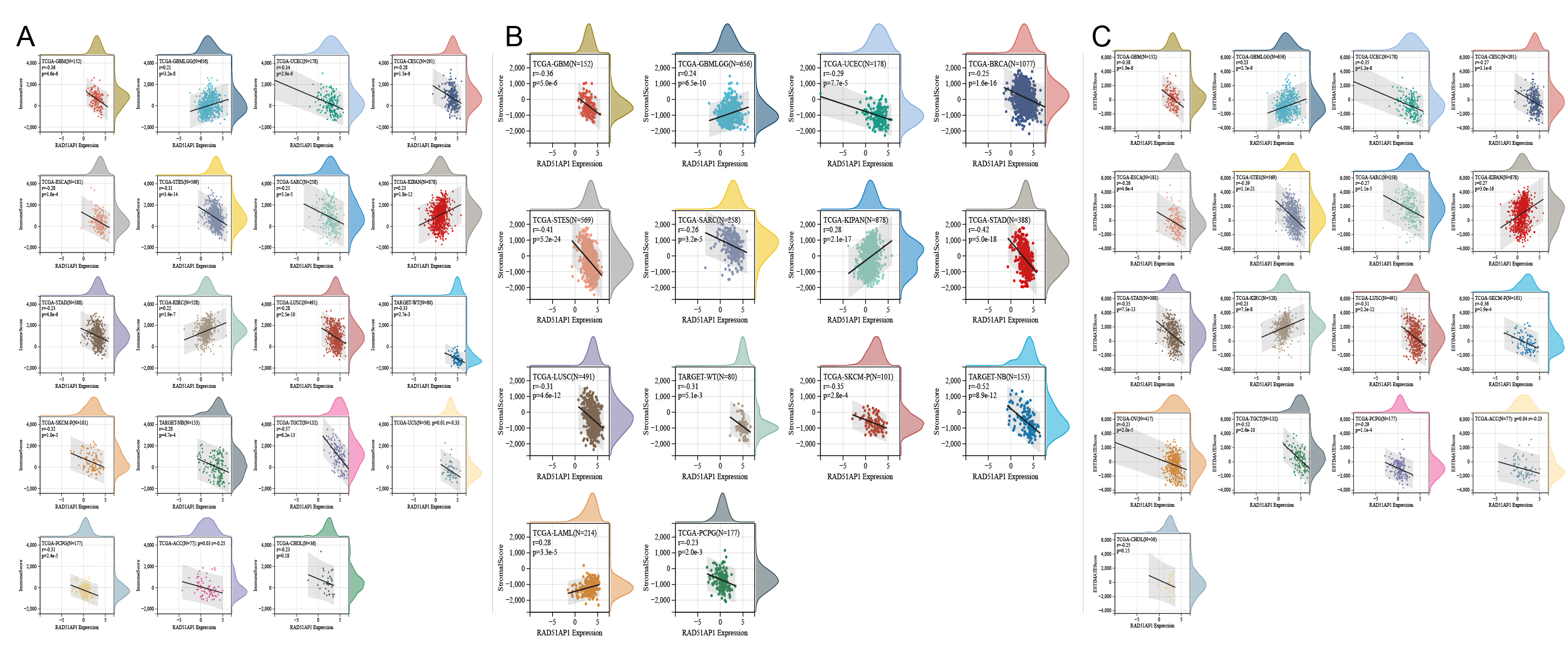


Forty-four cancer types and 10180 samples were analyzed via Estimation of STromal and Immune cells in MAlignant Tumours using Expression data (ESTIMATE) in R software. Both Immunescore **(A)** and ESTIMATEscore **(C)** were negatively correlated with RAD51AP1 expression in most cancers except GBMLGG, KIPAN, and KIRC. Stromalscore **(B)** was negatively correlated with RAD51AP1 expression in multiple cancers except for GBMLGG, KIPAN, and LAML.
